# *In vitro* Antibacterial Activity and Phytochemical Analysis of *Nicotiana tabacum* L. Extracted in Different Organic Solvents

**DOI:** 10.2174/1874285801711010352

**Published:** 2017-12-29

**Authors:** Gemechu Ameya, Aseer Manilal, Behailu Merdekios

**Affiliations:** 1 Department of Medical Laboratory Science, College of Medicine and Health Sciences, Arba Minch University, Arba Minch, Ethiopia; 2 Department of Public Health, College of Medicine and Health Sciences, Arba Minch University, Arba Minch, Ethiopia

**Keywords:** Antibacterial activity, *Nicotiana tabacum* L, Phytochemical analysis, Plant extract, Antibacterial property, Spectrum

## Abstract

**Background:**

Controlling infectious disease using medicinal plants is the oldest healthcare known to mankind. Regardless of the enormous advances observed in modern medicine, medicinal plants are still playing vital roles. However, only a small proportion of medicinal plants are examined for bioactive compounds which may vary in different factors. This study aimed to evaluate phytochemical constituent and antimicrobial activities of *Nicotiana tabacum* L. extracted by different solvents against three set of bacteria.

**Methods:**

*Nicotiana tabacum* L. was collected from the Western Ethiopia and extracted in seven organic solvents. An *in-vitro* anti-bacterial activity of plant extracts was carried out by agar well diffusion assay against microbial type culture collection of human pathogens, clinical bacterial isolates, and biofilm forming bacteria. Gas Chromatographic and Mass Spectroscopic (GC-MS) analysis was used to determine the phytochemical constituents.

**Results:**

Antimicrobial activities of plant extract vary by extraction solvents; and ethyl acetate based extracts showed better antimicrobial activities. Of the experimental organisms, biofilm forming uropathogens were the most sensitive while clinical isolates were quite resistant. Analysis of the active ethyl acetate extract by GC-MS evinced a mixture of five volatile compounds; and Pyridine, 3-(1-methyl-2-pyrrolidinyl)-, (S) was the major compound detected. The overall results of the present study revealed that *N. tabacum* L extract has high antimicrobial activities against biofilm forming uropathogens.

**Conclusion:**

High antimicrobial activity was observed in ethyl acetate extract of *N. tabacum* against the biofilm forming bacteria whereas the clinically isolated bacteria were the most resistant group. The antibacterial property demonstrated could be due to Pyridine, 3-(1-methyl-2-pyrrolidinyl)-(S) with a broad spectrum of activity.

## 1 INTRODUCTION

The decreasing efficacy of antibiotics and the upsurge of multidrug resistant pathogens are causing a great scourge to the global healthcare system and society [1]. Thence, a novel class of antimicrobials is urgently needed to combat the increased incidence of multidrug resistant pathogens [2]. It is proposed that about twenty novel classes of antibiotics are required to support modern medicine during the next 50 years [3].

Natural products from plants and animals have been the resource of most new classes of antibiotics so far [3]. In the study of natural antimicrobials, medicinal vegetal of terrestrial origin have long been a focus of attention. It was experimentally proved that plants are the sustainable source of diverse bioactive metabolites that can provide lead structures for development of novel drugs [4]. Now-a-days, the plant preparations successfully used in the treatment of many infectious diseases are envisaged for antimicrobial potency. However, their antimicrobial efficacy ascribed to the existence of diverse array of bioactive principles.

Regardless of the enormous sophistication occurring in the field of modern medicine, medicinal vegetal are still playing a pivotal role in the health care system [5]. During the past few decades, over 500,000 bioactive metabolites have been isolated from diverse terrestrial plants and many of them are utilized to design and develop new therapeutic agents in pharmaceutical firm [6]. Albeit, the plants are considered as a rich source of novel antimicrobial agents, these potential resources were hardly explored and majority of the bioactive compounds from the plants are yet to be discovered. Therefore, antimicrobial screening of medicinal plants provide the chance of discovering active candidates with new molecules of unique structure and potency.

*Nicotiana tabacum* L. is a perennial stout herbaceous plant, native to tropical and subtropical America and now cultivated worldwide as a cash crop. The officinal usage of *N. tabacum* has been extensively reviewed elsewhere [7]. In Ethiopian folkloric medicine, *N. tabacum* is widely used for managing both human and veterinary ailments such as cancer, ulcer, cough, snake bite, and respiratory tract infections. It also used as a vermifuge [8-10]. Antimicrobial potentiality of *N. tabacum* against diverse types of human and animal pathogens has been documented in some studies [11-13]. As yet however, antimicrobial activity and chemical analysis of *N. tabacum* in Ethiopia is not well studied [14]. Therefore, the present study is intended to evaluate the antimicrobial activity and GC-MS analysis of *N. tabacum* garnered from the Western Ethiopia.

## 2 MATERIALS AND METHODS

### 2.1 Study Design

An *in-vitro* experimental study of antibacterial activity using agar well diffusion method and Gas chromatography-mass spectrometric phytochemical analysis of *N. tabacum* leaves crude extracts was carried out. Positive and negative controls were used to monitor the antimicrobial activities of the extracts in all the assays.

### 2.2 Collection and Processing of Plant Materials

Fresh aerial leaves of *N. tabacum* L. was gathered from the Nekemte, Ethiopia. The area is 2,088m high above sea level with latitude and longitude of 9^0^5’N, and 36^0^ 33’E. The leaves were transported to the laboratory in a paper bag. Then sampled leaves were processed as already described elsewhere [5, 15]. Briefly, the leaves were preliminarily washed three times under running tap water and rinsed twice with sterile distilled water to remove the contaminants and other extraneous matter. The cleaned plant materials were cut into small pieces and air dried under shade for one week at room temperature to prevent photolysis and thermal degradation of metabolites. Completely dried leaves were weighed and ground finely using electric blender.

### 2.3 Extraction of Secondary Metabolites

The secondary metabolites of *N. tabacum* were extracted using seven organic solvents of increasing polarity specifically petroleum ether, chloroform, diethyl ether, ethyl acetate, acetone, dichloromethane and methanol [16]. The extraction with different organic solvents were carried out separately. Twenty grams of dried and finely powdered plant material was suspended in 200 ml of respective solvents to provide 10% w/v in 500 ml of sterilized screw capped bottles. The suspensions were placed at 35°C on an orbital shaker at 120 rpm for 48 hours to permit extraction of the active compounds. Subsequently, suspensions were filtered through Whatman (No. 1) filter paper and were evaporated to remove the excess solvent in rotary vacuum evaporator at 40 ºC (Yamato RE 801, Japan). The gummy residues of medicinal plant extract obtained from the seven different solvents were weighed, dissolved in deionized water and stored in deep freezer at -20 ºC until further use.

### 2.4 Test Microorganisms

Antimicrobial activity of secondary metabolites extracted from the *N. tabacum* were assessed using nine pathogenic bacteria precisely, three microbial type culture collection, four human multi-drug resistant clinical isolates, and two biofilm forming uropathogenic bacteria. The resistance patterns of clinical isolates were inspected using selective antibiotics. The clinical isolates and biofilm forming uropathogens were resistant against commonly used antibiotics such as chloramphenicol, tetracycline, erythromycin, clindamycin, amikacin, penicillin, ampicillin, nalidixic acid, and gentamicin. The biofilm forming potency of uropathogens were evaluated in our microbiology laboratory. All the experimental bacterial were grown in nutrient broth (Himedia®) at 37°C and maintained on nutrient agar slants at 4°C and sub-cultured prior to experimental use [17].

### 2.5 Antimicrobial Assay

The antibacterial assay was performed as per the methodology described in our previous studies [2, 15]. Fresh cultures of respective pathogens (approx. 10^6^ CFU/ml) were streaked over the surface of Mueller Hinton agar plates. In each triplicate of plates, cylindrical wells of 6mm diameter were punched using a sterile cork-hole borer. Appropriate organic extracts of 1 mg/ml were filled up to the brim of each well. The well with each solvent was used as negative controls. The plates were incubated at 37°C for 24 hours. After the period of incubation, zone of inhibition was measured by using sliding digital micro caliper; and antimicrobial activities of crude extracts were interpreted by calculating the area of inhibition zone around the well. The anti-biogram was statistically analyzed for the determination of skewness among the tested organisms using SPSS 20.0 software.

### 2.6 Determining the Mechanism of Antibiosis

The minimal inhibitory concentration (MIC) was determined by the broth dilution method as described elsewhere with slight modification [15, 17]. The highly sensitive strains including biofilm forming uropathogens such as *E. coli, Klebsiella* species and type culture *S. aureus* were used in determination of MIC. MICs were recorded as the lowest concentrations inhibiting visible growth. To measure the minimal bactericidal concentrations (MBC), the MIC cultures were plated on fresh Mueller-Hinton agar and incubated for 24 h at 37°C. A reduction of at least 99% of the colonies, compared with the culture of the initial inoculum of the strains, was regarded as evidence of bactericidal activity. When the ratio of MBC/MIC was ≤2, the extract was considered as bactericidal; otherwise it was considered bacteriostatic. If the ratio was ≥16, the extract was considered as ineffective.

### 2.7 Gas Chromatographic and Mass Spectroscopic (GC-MS) Analysis

The ethyl acetate extract of *N. tabacum* that showed broad spectrum of activity was subjected to GC-MS analysis. The organic extract was chemically analyzed using a Shimadzu QP-2010 Gas Chromatograph equipped with mass spectrometer and a capillary column of inner diameter 0.25 mm and it was used with helium at 1 ml min^-1^as a carrier gas. GC oven temperature was kept at 100°C for two minutes, programmed to 250°C at the rate of 10°C min^-1^and then kept unceasing for 13 minutes. The split ratio was adjusted to 1: 25 and the injection volume was 2 µl. The injection and detector temperature was 200°C. The GC-MS electron ionization mode was 150 eV. The mass scan range was from m/z 40 to 400 amu. Peak identification was carried out using NIST Version 2.0 (2005).

### 2.8 Data Analysis

The mean and standard deviation of the measurement was used to present the findings of the agar well diffusion assay. The difference among mean values were assessed using one way analysis of variance (ANOVA) using SPSS version 20 (Statistical Package for Social Services, Chicago, IL, USA).

## 3 RESULTS

Extracted *Nicotiana tabacum* L. showed various range of antimicrobial activities against the three sets of bacteria. The overall activity index of the seven solvent extracts of *N. tabacum* against different panel of tested bacteria is appended in Table (**1**). Of the seven solvents used, ethyl acetate based extract showed significantly high (P<0.05) antimicrobial activity followed by methanol and dichloromethane. The ethyl acetate extract of *N. tabacum* displayed broad spectra of activity and it was efficiently subdued the growth of all the tested pathogens. However, under this experimental condition, no activity was manifested in petroleum ether, chloroform, diethyl ether, and acetone extract at 1mg/ml concentration of the extracts. The results of the present study indicated that organic solvents have significant influence on the extraction of antimicrobial principles.

**Fig. (1):**
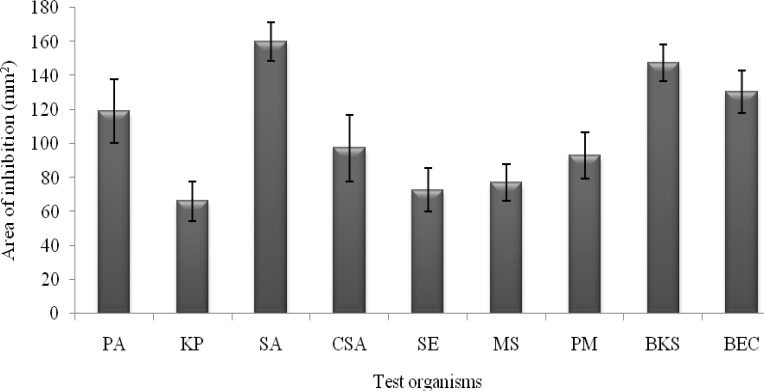
Antibacterial potential of ethyl acetate extract of *N. tabacum* L. The activity index was calculated as mm^2^ area based on the diameter of halo displayed. [PA- *P. aeruginosa*; KP- *K. pneumonia*; SA- *S. aureus*; CSA- clinical *S. aureus;* SE- *Salmonella enterica* subsp. *enteric* serotype Typhi; MS- *Micrococcus* sp.; PM- *P. mirabilis*; BKS- biofilm forming *Klebsiella* sp.; BEC- Biofilm forming *E. coli*

The inhibitory spectra of the medicinal plant extracts against the human type culture strains ranged between 66.29±11.61 mm^2^ to 159.9 ±11.31 mm^2^ Fig. (**1**). The highest inhibitory value of 159.9±11.31 mm^2^ was extended against the *S. aureus* followed by 119.23 ± 18.7mm^2^ which was against *P. aeruginosa.* However, the inhibitory potential was meager against *K. pneumonia* (66.29 ± 11.61mm^2^). In the case of tested clinical isolates, highest inhibitory value of 97.41 ± 19.62 mm^2^ was exercised against *S. aureus*; however, the Gram negative pathogen, *Salmonella enteric* subsp. *enteric* serotype Typhi (72.8 ± 12.9 mm^2^) was found to be quite resistant. Concerning the tested biofilm forming uropathogens, the inhibitory area was 130.72 ± 12.5 and 147.5 ± 10.82 mm^2^ against *E. coli* and *Klebsiella* species respectively (Fig. **1**).

The results of minimum inhibitory concentration (MIC) and minimum bactericidal concentration (MBC) of the highly sensitive strains including biofilm bacteria *E. coli, Klebsiella* species, and *S. aureus* were performed and indicated in Table (**2**). The minimum inhibitory concentration of the medicinal plant extracts against biofilm forming uropathogens and type culture ranged from 62.50 to 500 (µg/ml) and the concomitant MBC values ranged from 125 to 1000 (µg/ml). On the basis of preliminary findings, the ethyl acetate extract of *N. tabacum* was further selected for chemical investigation by GC-MS analysis.

### 3.1 GC- MS Analysis of Ethyl Acetate Extract of *N. tabacum*

In order to determine the chemical constituents responsible for antimicrobial activity, the crude extract of ethyl acetate of *N. tabacum* was subjected to GC-MS analysis. The preliminary GC-MS analysis of *N. tabacum* on the basis of spectral data revealed the presence of a mixture of volatile compounds. A total of five prominent peaks were observed with retention times as summarized in Fig. (**2**). The prevailing phytochemical constituents detected are 3, 4, 5, 6-Tetrahydro-1, 3-dimethyl-2(1h)-pyrimidinone, Pyridine, 3-(1-methyl-2-pyrrolidinyl)-, (S)-, Isododecane, n-pentadecane, and Tetradecylaldehyde is presented in Table (**3**). Among detected phytochemicals of the medicinal plant extracts, 3-(1-methyl-2-pyrrolidinyl)-, (S) was the major ingrediant.

## 4 DISCUSSION

In the present study, *N. tabacum* collected from the western Ethiopia prefecture was subjected to extraction process using seven organic solvents of increasing polarity. The resultant crude extracts were then inspected for antimicrobial activity against three sets of pathogenic bacteria such as human type cultures, clinical isolates and biofilm forming bacteria. The overall results of antimicrobial activity of *N. tabacum* extracted using different organic solvents have been expressed in terms of the area of zone of inhibition in millimeter square. The crude extracts that produced zone of inhibition ≥28 mm^2^ were considered as active [17]. The MBC/MIC ratio was determined to identify whether the extract was a bactericidal or a bacteriostatic in nature. Since the MBC/MIC ratio is ≤2, the extract can be considered as bactericidal agent [17].

The secondary metabolites of plant constituents are sustainable resource for exploring novel antibiotics [15]. The secondary metabolites isolated from plants can repress the growth of human bacterial pathogens by mechanisms that may differ from antibiotics currently in use [18]. Evaluation of antimicrobial activities can be considered as effective indicator to analyze the capability of the plant species to synthesize antibiotic metabolites. Studies have documented the antimicrobial activity of *N. tabacum* from various locales [13, 19-21]. However, literature pertaining to the exploration of antimicrobial activity of *N. tabacum* from Ethiopia is scanty [14].

The finding of present study exhibited that, crude extracts of the *N. tabacum* showed antimicrobial activity in different ranges with respect to the solvents and test organisms. These differences in efficacy at the *in vitro* level could be linked to the level of active substances in the respective solvent extracts or due to the virulence of bacteria. Of the seven solvent tested, ethyl acetate was found to be the best solvent for extraction of antimicrobial secondary metabolites from dried plant material followed by methanol and dichloromethane. The activity demonstrated by the extracts of other solvents may be due to varying degrees of solubility for antimicrobial constituents in the medicinal plant [16].

The extracts of *N. tabacum* obtained using ethyl acetete effeciently impeded the growth of all the tested pathogens in varing degrees. In general, it can be opined that the activity of ethyl acetete extracts was more evident against the biofilm forming uropathogens while the clinical isolates were the most resistant group. On the other hand, human type cultures were found to be moderately susceptible except in the case of *K. pneumonia*. In accordance with the present study, ethyl extract of *N. tabacum* exerts significant antibacterial activity against Gram positive and Gram negative bacteria. Study conducted in Pakistan showed that *N. tabacum* has a maximum zones of inhibition against *S. aureus* at 900mg/ml concentration while *E. coli* and *Pseudomonas aeruginosa* showed less susceptible to the extract [13].

In this study, ethyl acetate extarct of *N. tabacum* was analysed for phytochemicals by GC-MS. It is envisaged that the antibacterial property demonstrated by the ethyl acetete extract of *N. tabacum* against different bacterial strains could be due to chemical entity (Pyridine, 3-(1-methyl-2-pyrrolidinyl)-, (S)) with a broad spectrum antimicrobial activity [22-24] or could be related with synergistic activity of different chemical entities, since antibacterial activities are pertained to the presence of secondary metabolites. Another studies also showed that *N. tabacum* contains potent antibacterial secondary metabolites [25, 26].

In western part of Ethiopia, *N. tabacum* L. is a well known medicinal plant that is used for human and veterinary disease treatment. People rely on this medicinal plant because it is culturally integrated, easily available and affordable. The juice of the plant is used to treat local skin infection and mastitis. Studies also show that local people use it for treatment of blackleg which is a condition presented with swelling of thigh, muscular damage of involved legs and for gastrointestinal tract disease of animals [9, 14]. For the veterinary use, dried leaves are soaked overnight with water, squeezed and given in one cup at a time [9]. The study conducted in Pakistan also showed that extract of leaves is applied to treat ectoparasite [27]. Therefore, currently there is high utilization of this medicinal plant by the local people of Ethiopia even though there is no strong scientific proof. Taking this into consideration, this study tried to show the antimicrobial effect of the medicinal plant against infectious pathogens which is traditionally applied by local practitioners.

## CONCLUSION

The ethyl acetate extract of *N. tabacum* L. displayed broad spectra of activity and it efficiently subdued the growth of all the tested pathogens. High antimicrobial activity of ethyl acetete extract of *N. tabacum* was observed against the biofilm forming bacteria while the clinical isolates were the most resistant group. The antibacterial property demonstrated by the ethyl acetete extract of *N. tabacum* against different bacterial strains could be due to chemical entity (Pyridine, 3-(1-methyl-2-pyrrolidinyl)-, (S)) with a broad spectrum of activity. Further bioassay guided fractionation and purification of *N. tabacum* is recommended to determine antimicrobial effect of specific compounds.

## Figures and Tables

**Fig. (2):**
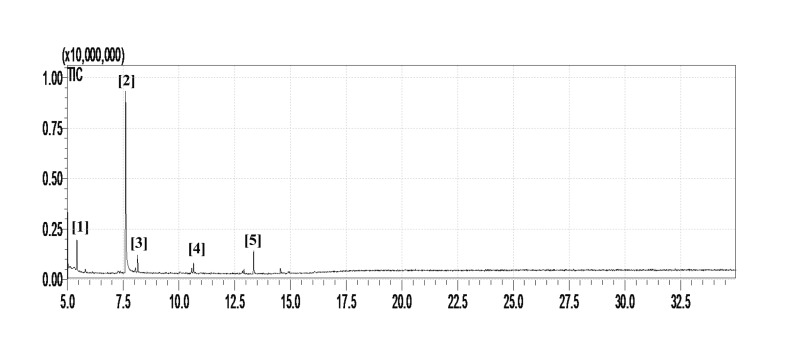
Gas Chromatography-Mass Spectrometry chromatogram of the ethyl acetate extract of *N. tabacum* [1] 3,4,5,6-Tetrahydro-1,3-dimethyl-2(1h)-pyrimidinone; [2] Pyridine, 3-(1-methyl-2-pyrrolidinyl)-, (S); [3] Isododecane; [4] n-Pentadecane; [5] Tetradecylaldehyde.

**Table 1:** Overall activity index of different solvent extracts of *N. tabacum* against different panel of test bacteria.

Solvents	**% of Antimicrobial Activity**		
	**Human Pathogens** **(ATCC)**	**Clinical Isolates**	**Biofilm Forming Uropathogens**
Petroleum ether	0	0	0
Chloroform	0	0	0
Diethyl ether	0	0	0
Ethyl acetate	100	100	100
Acetone	0	0	0
Dichloromethane	33.3	25	50
Methanol	66.6	75	100

*Zone of Inhibition ≥ 28 mm^2^ was considered as active.

**Table 2:** Minimum inhibitory concentration of *N. tabacum.*

Test Strain	MIC (µg/ml)	MBC (µg/ml)	MBC/MIC
Biofilm forming *E. coli*	62.50	125	2
Biofilm forming *Klebsiella* sp.	125	125	1
*S. aureus*	500	1000	2

**Table 3:** Components identified in the ethyl acetate extract of *N. tabacum* by GC-MS study.

No.	RT	**Major Compounds**	**Identified Structure**	**Molecular Weight**	**Chemical Formula**
1	5.40	3,4,5,6-Tetrahydro-1,3-dimethyl-2(1h)-pyrimidinone	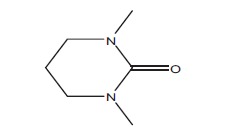	128.17	C_6_H_12_N_2_O
2	7.57	Pyridine, 3-(1-methyl-2-pyrrolidinyl)-, (S)	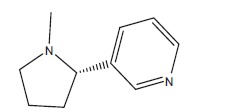	162.23	C_10_H_14_N_2_
3	8.11	Isododecane	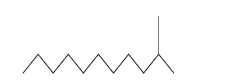	170.33	C_12_H_26_
4	10.65	n-Pentadecane	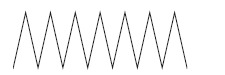	212.41	C_15_H_32_
5	13.14	Tetradecylaldehyde	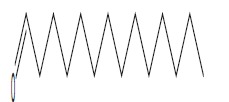	212.37	C_14_H_28_O

RT: Retention Time

## LIST OF ABBREVIATIONS

ATCC: American type culture collection;
CFU: Colony forming units;
GC-MS: Gas chromatographic and mass spectroscopic;
MBC: Minimal bactericidal concentrations;
MIC: Minimal inhibitory concentration;
NIST: National institute of standards and technology;
SPSS: Statistical package for the social sciences
